# Diclofenac Resensitizes Methicillin‐Resistant *Staphylococcus aureus* to *β*‐Lactams and Prevents Implant Infections

**DOI:** 10.1002/advs.202100681

**Published:** 2021-05-03

**Authors:** Shutao Zhang, Xinhua Qu, Haozheng Tang, You Wang, Hongtao Yang, Weien Yuan, Bing Yue

**Affiliations:** ^1^ Department of Bone and Joint Surgery, Department of Orthopedics, Renji Hospital, School of Medicine Shanghai Jiaotong University Shanghai 200127 China; ^2^ Department of Plastic & Reconstructive Surgery The Ohio State University Columbus OH 43210 USA; ^3^ School of Medical Science and Engineering Beihang University Beijing 100191 China; ^4^ Engineering Research Center of Cell & Therapeutic Antibody Ministry of Education School of Pharmacy Shanghai Jiao Tong University Shanghai 200240 China

**Keywords:** diclofenac, methicillin‐resistant *Staphylococcus aureus*, oxacillin, perioperative infection

## Abstract

Implant infections caused by methicillin‐resistant *Staphylococcus aureus* (MRSA) can cause major complications during the perioperative period. Diclofenac, one of the most widely used nonsteroidal anti‐inflammatory drugs, is often used to relieve pain and inflammation. In this study, it is found that high‐dose diclofenac can inhibit the growth of MRSA, and does not easily induce drug‐resistant mutations after continuous passage. However, low‐doses diclofenac can resensitize bacteria to *β*‐lactams, which help to circumvent drug resistance and improve the antibacterial efficacy of conventional antibiotics. Further, low‐dose diclofenac in combination with *β*‐lactams inhibit MRSA associated biofilm formation in implants. Transcriptomic and proteomic analyses indicate that diclofenac can reduce the expression of genes and proteins associated with *β*‐lactam resistance: mecA, mecR, and blaZ; peptidoglycan biosynthesis: murA, murC, femA, and femB; and biofilm formation: altE and fnbP. Murine implant infection models indicate that diclofenac combined with *β*‐lactams, can substantially alleviate MRSA infections in vivo. In addition, it is investigated that low dose diclofenac can inhibit MRSA antibiotic resistance via the mecA/blaZ pathway and related biofilms in implants. The synergistic effect of diclofenac and *β*‐lactams might have promising applications for preventing perioperative infection, considering its multitarget effects against MRSA.

## Graphical abstract

Diclofenac enhances the sensitivity of methicillin‐resistant *Staphylococcus aureus* toward the *β*‐lactam antibiotics, which help to circumvent drug resistance and improve the antibacterial efficacy of conventional antibiotics. Besides, murine implant infection models show that diclofenac combined with oxacillin, can alleviate MRSA infections. Mechanistic studies indicate that diclofenac can inhibit MRSA antibiotic resistance through the MecA/BlaZ pathway.

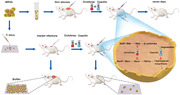

## 1 Introduction

With the ever‐growing global elderly population, many medical implant devices have been developed to improve quality of life, including joint prostheses and catheters. However, the increasing application of medical implants also increases the incidence of postoperative infection.^[^ ^1^ ^]^ In fact, an estimated 25.6% of all healthcare‐related infections in the United States are attributed to device‐associated infections.^[^ ^2^ ^]^ Specifically, patients with weakened immune systems, including those with diabetes, rheumatoid arthritis, malnutrition, and others, are generally at higher risk for developing implant‐related infections. Moreover, clinical infections often require long‐term antibiotic treatment and multiple surgical debridement procedures, which can have significant detrimental effects for the patients.^[^ ^3^ ^]^ In addition, methicillin‐resistant *Staphylococcus aureus* (MRSA), which is resistant to most *β*‐lactams, including oxacillin and has increasing global prevalence, can also cause chronic infection.^[^ ^4^ ^]^ Currently, although various precautionary measures have been established to reduce the incidence of infections, including performing surgeries under laminar flow air conditions and administering antibiotics prophylactically, the occurrence of postoperative infections remains high.^[^ ^5^ ^]^

Moreover, several factors have been reported as being associated with the treatment failure of implant‐related infections. First, implant surfaces can provide shelter for bacteria to survive.^[^ ^6^ ^]^ In fact, fewer bacterial cells are required to cause infections following implant‐related surgeries compared to those not involving placement of indwelling devices.^[^ ^7^ ^]^ Second, the formation of biofilms on the surface of implants serves to protect bacteria, altering their growth state while reducing antibiotic penetration. Hence, biofilm formation can facilitate the development of drug‐resistant bacteria by limiting their contact with antimicrobial substances.^[^ ^8^ ^]^ Third, the prevalence of MRSA, which exhibits broad‐spectrum resistance to *β*‐lactams, poses a significant threat to patients as effective antibiotics are rarely available.^[^ ^9^ ^]^ Fourth, the presence of medical implants, and the associated tissue damage that occurs during surgery, can interfere with normal immune responses. Finally, postoperative pain is also recognized as a risk factor for infections as it can reduce wound perfusion and decrease tissue oxygen partial pressure, thus impairing the ability of immune cells to induce oxidative killing.^[^ ^10^ ^]^ Taken together, these factors enable various opportunistic pathogens to replicate rapidly causing postoperative infections.^[^ ^11^ ^]^

Recently, drug repurposing and synergistic drug screening have become promising approaches to combat infections caused by multidrug‐resistant pathogens.^[^ ^12^ ^]^ Compared with the development of novel therapeutic agents, this strategy is considered more efficient for the treatment of severe infections as it has the potential to overcome challenges associated with weak activity elicited by individual drugs.^[^ ^13^ ^]^ For example, statins have been shown to disassemble bacterial membrane microdomains, thereby decreasing antibiotic resistance and resensitizing MRSA to antibiotic therapies.^[^ ^14^ ^]^ Similarly, norgestimate has been reported to inhibit staphylococcal biofilm formation and resensitize MRSA to *β*‐lactams.^[^ ^15^ ^]^ Although these drugs can help to control MRSA replication, associated side effects have also been reported for some patients.^[^ ^16^ ^]^ Besides, the efficacy of these drugs for preventing implant infections remains unclear. It is, therefore, necessary to identify a promising therapeutic regimen to target drug‐resistant bacteria that can be readily administered in clinical settings.

Nonsteroidal anti‐inflammatory drugs (NSAIDs) represent a group of widely used analgesic and antipyretic agents.^[^ ^17^ ^]^ In the United States alone, over 111 million NSAID prescriptions are administered with approximately $2 billion spent on over‐the‐counter NSAIDs each year.^[^ ^18^ ^]^ Diclofenac is a commonly used NSAID that inhibits the synthesis of prostaglandins by inactivating cyclooxygenase.^[^ ^19^ ^]^ As it is a well‐tolerated NSAID, with few reported side effects, diclofenac is considered one of the few “first choice” drugs for the treatment of painful conditions such as postoperative pain, and inflammatory conditions.^[^ ^20^ ^]^ Additionally, several researchers suggest that diclofenac could inhibit the proliferation of a broad spectrum of microorganisms, including *Escherichia coli*, *S. aureus*, *Candida albicans*, *Listeria monocytogenes*, and *Mycobacterium tuberculosis*.^[^ ^21^ ^]^ Moreover, antibacterial effects have also been noted following administration of diclofenac in combination with *β*‐lactams, which are often used during the perioperative period. However, the mechanism by which diclofenac resensitizes MRSA to *β*‐lactams has not been systematically reported.

During the perioperative period, *β*‐lactams are generally recommended as the first‐line drug to prevent implant infections.^[^ ^22^ ^]^ However, its application does not effectively prevent drug‐resistant infections. Meanwhile, NSAIDs are often used intraoperatively in combination with analgesic agents for local infiltration to relieve postoperative pain following joint arthroplasty.^[^ ^23^ ^]^ Hence, these two perioperative drugs may function synergistically to protect against the colonization and replication of drug‐resistant bacteria.

**Figure 1:**
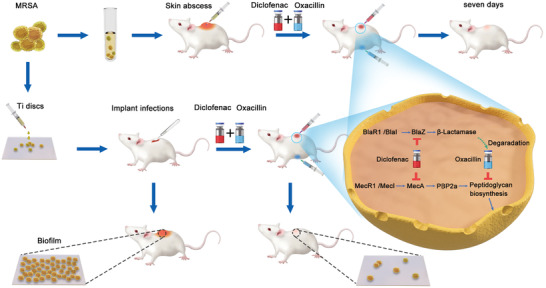
Scheme illustration of diclofenac used in combination with oxacillin to alleviate implant infections caused by MRSA. The proposed mechanism suggests that diclofenac can enhance the bactericidal activity of oxacillin by inhibiting the expression of *β*‐lactamase and PBP2a.

In this study, we conducted a series of in vitro and in vivo experiments (**Figure** **1**) to explore whether diclofenac can inhibit the growth of multidrug‐resistant staphylococci; to determine whether low dose diclofenac can resensitize MRSA to *β*‐lactams, such as oxacillin and if so, to elucidate the underlying mechanism; and to determine whether low dose diclofenac can be used in combination with *β*‐lactams to reduce implant‐related infections.

## 2 Results

### 2.1 Diclofenac Inhibits the Growth of Methicillin‐Resistant Staphylococci

**Figure 2:**
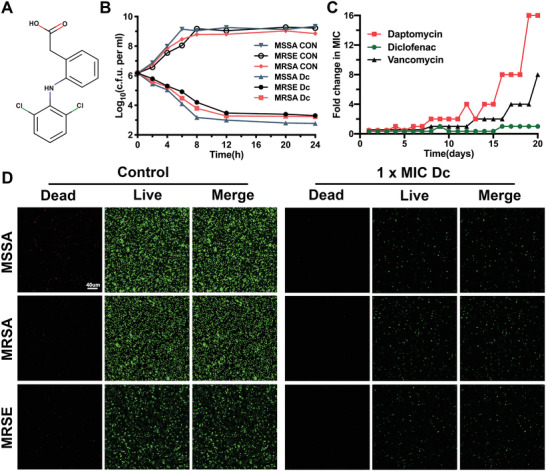
Diclofenac inhibits the growth of methicillin‐resistant staphylococci. A) Chemical structure of diclofenac. B) Time‐dependent killing of methicillin‐sensitive *Staphylococcus aureu*s (MSSA) ATCC 25923, MRSA ATCC 43300 and methicillin‐resistant *Staphylococcus epidermidis* (MRSE) ATCC 35984 by diclofenac at the minimal inhibitory concentration (MIC). The detection limit of the experiment was ≥ 10^2^ CFU mL^−1^. CON, Control; Dc, Diclofenac. Each point represents the average value of three independent trials. Error bars are not presented for clarity. C) Resistance development during serial passaging in the presence of sub‐MIC concentrations of antimicrobials. Daptomycin and vancomycin served as the positive control. Each point represents the average value of three independent trials. D) Representative confocal laser scanning microscopy (CLSM) images of MSSA, MRSA, and MRSE treated with 1× MIC diclofenac and stained for detecting bacterial viability.

The chemical structure of diclofenac is shown in **Figure** **2**A. To examine the antibacterial effect of diclofenac against drug‐resistant Staphylococcus, we performed a minimum inhibitory concentration (MIC) assay, time kill assay, and Live/Dead bacteria staining. The MIC value of diclofenac (125 µg mL^−1^) was found to be the same for MRSA, methicillin‐sensitive *Staphylococcus aureus* (MSSA), and methicillin‐resistant *Staphylococcus epidermidis* (MRSE). Moreover, bacterial viability was found to significantly decline after treatment with diclofenac at the MIC (Figure 2B). Additionally, it is important that antibiotics possess a low resistance frequency. In this regard, diclofenac was found to induce lower levels of resistance compared to daptomycin and vancomycin (Figure 2C), which is commonly administered as a primary agent for the treatment of *MRSA* infection.^[^ ^24^ ^]^ Moreover, compared with the control group, the number of viable bacteria in the diclofenac treatment group was lower (Figure 2D).

### 2.2 Diclofenac Inhibits the Expression of Genes Associated with *β*‐Lactam Resistance and Biofilm Formation

**Figure 3:**
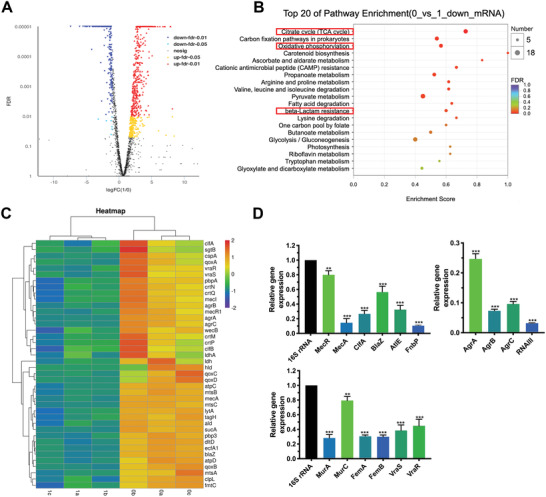
Changes in the transcriptome of MRSA ATCC 43300 treated with diclofenac (62.5 µg mL^−1^) or DMSO (control). A) Volcano map for the distribution of differentially expressed genes (DEGs). B) DEGs enriched in the KEGG pathway. Entries with larger bubbles contain more DEGs. C) Cluster analysis of selected DEGs. Red indicates a highly expressed gene, and blue indicates a weakly expressed gene. Each group contains data for three independent samples. D) Validation of RNA‐Seq data for selected genes by qRT‐PCR. The expression of DEGs involved in *β*‐lactam resistance, peptidoglycan biosynthesis, quorum sensing, and biofilm formation was evaluated. Data analysis was performed using the comparative CT method, with 16S rRNA serving as the comparator. The results are presented as fold‐changes relative to the control, which was set to a value of 1. Data are expressed as the mean ± SD; *p*‐values are calculated using one‐way ANOVA with Dunnett correction. *n* = 3; ** *p* < 0.01; *** *p* < 0.001.

The differentially expressed genes (DEGs) of MRSA were statistically analyzed to elucidate the antibacterial mechanism of diclofenac. We first applied a quantile normalization to the fragment per kilobase per million mapped reads (FPKM) values (Student's *t*‐test at a *p* value = 0.05), followed by selection of candidate genes displaying differential expression of at least two‐fold compared to that in the control group. A total of 996 DEGs (678 upregulated and 318 downregulated) with highly significant expression patterns before and after diclofenac treatment were identified (**Figure** **3**A). Enrichment analysis of KEGG pathways showed that DEGs associated with *β*‐lactam resistance and energy metabolism were significantly downregulated by diclofenac (Figure 3B). All the sequencing data generated in this study have been submitted to the NCBI Sequence Read Archive with accession number SRP297471.

Generally, penicillin‐binding protein 2a (PBP2a) shows low *β*‐lactam affinity and remains active to allow peptidoglycan biosynthesis at normally lethal *β*‐lactam concentrations.^[^ ^25^ ^]^ As shown in Figure 3C, *mecA*, which is the primary resistance gene encoding PBP2a, was reduced by 3.07‐fold upon treatment with diclofenac compared to that in the control group. Its inactivation not only results in a poorly crosslinked peptidoglycan and *β*‐lactam hyper‐susceptibility but is also associated with increased susceptibility to other antibiotics.^[^ ^26^ ^]^ Besides, diclofenac induces a significant 2.29‐ and 2.02‐fold reduction in the levels of *VraS* and *VraR*, respectively, which are central to peptidoglycan synthesis.^[^ ^27^ ^]^ In addition, the fem factors (essential for methicillin resistance),^[^ ^28^ ^]^ *femA* and *femB*, were also reduced by 1.31‐ and 1.65‐fold, respectively, in the diclofenac‐treated group compared to the control group.

Another important pathway inhibited by diclofenac is quorum sensing via the accessory gene regulator (*agr*) system. The *agr* phenotype may affect several aspects of biofilm behavior, including attachment to surfaces and biofilm dispersal and may contribute to the chronicity of biofilm‐associated infections.^[^ ^29^ ^]^ The RNA‐seq results indicated that *agrA*, *B*, and *C* were significantly reduced by 5.50‐, 3.87‐, and 5.22‐fold, respectively, in the diclofenac‐treated group compared to the control group. Meanwhile, *hld*, whose 3ʹ end encodes delta toxin, was also reduced by 7.44‐fold.

qRT‐PCR assay was further conducted to validate transcriptional changes induced by diclofenac. Genes associated with *β*‐lactam resistance, peptidoglycan biosynthesis, biofilm formation, and quorum sensing were carefully examined. A relatively high consistency between qRT‐PCR and transcriptomic data was observed in terms of expression patterns (Figure 3D). Although the absolute fold change for each gene differed between the qRT‐PCR and transcriptomic data, similar trends were observed.

### 2.3 Diclofenac Restores *β*‐Lactams Efficacy toward MRSA

**Figure 4:**
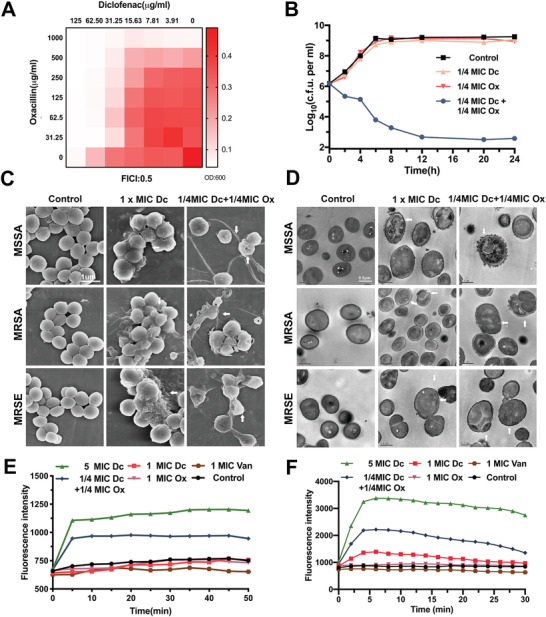
Diclofenac combined with oxacillin destroys the ultrastructure of bacteria. A) Synergism between diclofenac and oxacillin, evaluated against MRSA ATCC 43300 using the fractional inhibitory concentration index (FICI) microdilution checkerboard method. OD_600_ nm was measured after 18‐h incubation at 37 °C. Experiments were independently repeated twice. Synergy, FICI ≤ 0.5; no interaction, 0.5 < FICI ≤ 4; antagonism, FICI > 4. B) Time‐dependent killing of MRSA by combination of 1/4 MIC diclofenac (Dc) and 1/4 MIC oxacillin (Ox). The detection limit of the experiment was ≥ 10^2^ CFU mL^–1^. Each point represents the average value of three independent trials. Error bars are not presented for clarity. C) Scanning electron microscopy images of bacteria incubated with 1× MIC diclofenac (125 µg mL^−1^), 1/4xMIC diclofenac (Dc), and 1/4× MIC oxacillin (Ox) for 24 h. D) Transmission electron microscopy images of bacteria incubated with 1× MIC diclofenac, 1/4 MIC diclofenac (Dc), and 1/4 MIC oxacillin (Ox) for 8 h. White arrowheads indicate bacteria with damaged membranes and walls. The experiment was repeated three times. E) Membrane permeabilization analysis. Uptake of propidium iodide by MRSA treated with diclofenac (Dc), oxacillin (Ox) or vancomycin (Van). Each point represents the average value of three independent trials. Error bars are not presented for clarity. F) Membrane depolarization assay. Changes in fluorescence intensity of the membrane potential‐sensitive dye diSC3(5) within 30 min after treatment of MRSA with diclofenac, oxacillin or vancomycin. Each point represents the average value of three independent trials. Error bars are not presented for clarity.

Combined drug therapy to combat MRSA infections is often effective for reducing the associated side effects of individual agents.^[^ ^12^ ^]^ Therefore, the synergistic effects of drugs are important considerations. As shown in **Figure** **4**A and Table S2 in the Supporting Information, low dose diclofenac significantly enhanced the antibacterial effect of several *β*‐lactams. Specifically, 1/4 MIC of diclofenac (31.25 µg mL^−1^) and 1/4 MIC of oxacillin (250 µg mL^−1^) effectively inhibited MRSA growth. Meanwhile, the fractional inhibitory concentration index (FICI) was 0.5 for oxacillin, indicating that diclofenac is an efficient compound to offset drug resistance. Additionally, the time kill assay results demonstrated that a combination of 1/4 MIC diclofenac and 1/4 MIC oxacillin could significantly inhibit the growth of MRSA (Figure 4B). However, 1/4 MIC oxacillin or 1/4 MIC diclofenac alone was not effective at inhibiting MRSA growth.

### 2.4 Diclofenac Combined with Oxacillin Destroys the Ultrastructure of Bacteria

Electron micrographs obtained for the control group showed regular, intact surfaces, as expected (Figure 4C). In contrast, cells treated with low dose of diclofenac and oxacillin displayed severe cellular damage, including irregular and collapsed surfaces. These results were then confirmed using transmission electron microscopy (TEM), which demonstrated that untreated cells exhibited a circular and smooth shape, surrounded by a well‐defined cell wall, with a prominent septal midline within the nascent septum (Figure 4D). In contrast, diclofenac‐treated bacteria presented irregular thickening and an increase in the occurrence of “fuzzy” cell walls.

The membrane permeability was then measured using propidium iodide (PI), which is incorporated into cells with damaged membranes (Figure 4E). Low dose of diclofenac combined with oxacillin was found to significantly reduce the membrane integrity. Similar results were observed in the 5× MIC diclofenac group. However, similar phenomena were not observed in the 1× MIC diclofenac group and the 1× MIC vancomycin group. Membrane depolarization was determined using the potential‐sensitive fluorescent dye 3,3‐dipropylthiocarbocyanine diSC3(5), which is released into the medium following disruption of membrane potential. Low dose of diclofenac combined with oxacillin was found to significantly dissipate the membrane potential in the MRSA group compared to the control group (Figure 4F). Additionally, a cellular ATP level assay and efflux inhibition assay were conducted to validate the RNA‐seq results. As shown in Figure S1A,B in the Supporting Information, low dose diclofenac combined with oxacillin significantly reduced the levels of cellular ATP compared to oxacillin. This inhibitory effect occurred in a dose‐dependent manner. However, the efflux pump activity was not significantly affected by diclofenac treatment.

### 2.5 Diclofenac Combined with Oxacillin Prevents Biofilm Formation

**Figure 5:**
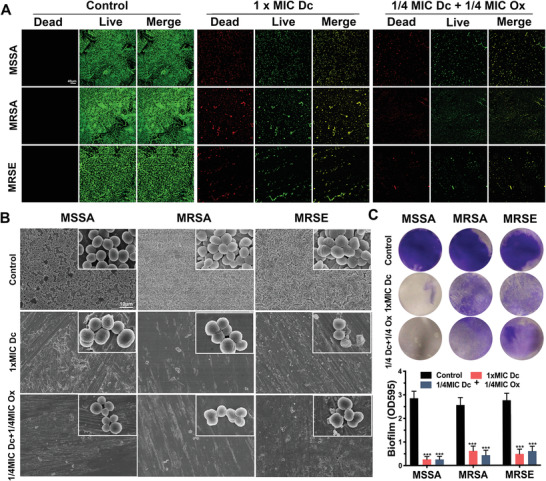
Diclofenac combined with oxacillin prevents biofilm formation. A) Confocal laser scanning microscopy images of biofilms treated with 1× MIC diclofenac (Dc) alone or 1/4 MIC diclofenac (Dc) in combination with 1/4 MIC oxacillin (Ox) for 24 h and stained by using a bacterial viability kit. B) Scanning electron microscopy images of biofilms treated with 1× MIC diclofenac (Dc) alone or 1/4 MIC diclofenac (Dc) in combination with 1/4 MIC oxacillin (Ox) for 24 h. C) Biofilms treated with 1× MIC diclofenac (Dc) alone or 1/4 MIC diclofenac (Dc) in combination with 1/4 MIC oxacillin (Ox) for 24 h and stained with 1% crystal violet. Biofilm formation was quantified by measuring sample absorbance at 595 nm using a microtiter plate reader. Data are expressed as the mean ± SD; *n* = 5; *p*‐values are calculated using one‐way ANOVA with Dunnett correction; *** *p* < 0.001.

Bacterial adherence on the surface of Ti6Al4V disks was observed by CLSM (**Figure** **5**A). All control group samples were well coated with biofilm. In contrast, the diclofenac treatment groups exhibited less colonization of bacteria. Next, the morphology of biofilms was observed with scanning electron microscopy (SEM). The biofilm in the control group was composed of several compact bacterial colonies (Figure 5B). However, there was less adherence of bacteria on the surface of Ti6Al4V disks following treatment with low doses of diclofenac and oxacillin. Next, crystal violet staining indicated that high dose of diclofenac, or the combination of low dose of diclofenac and oxacillin, significantly reduced the biofilm mass (Figure 5C).

### 2.6 Diclofenac Leads to Proteomic Alterations in MRSA

**Figure 6:**
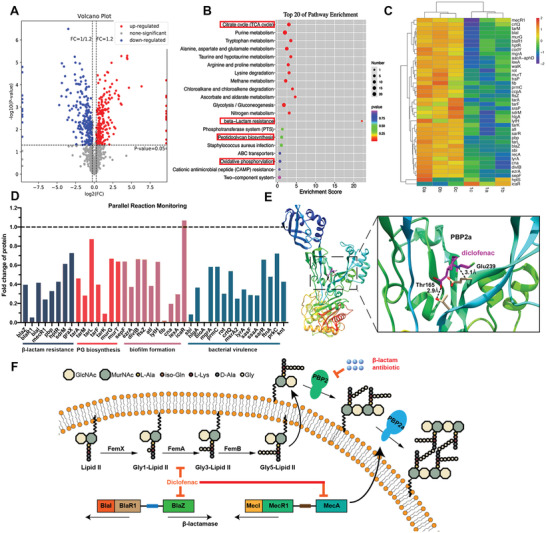
Diclofenac causes MRSA proteomic alterations. A) Volcano plot for screening of differentially expressed proteins (DEPs) between the control group and diclofenac‐treated groups. B) DEPs enriched in the KEGG pathway. The ordinate is the top 20 pathways with significant enrichment. C) Heat map of hierarchical clustering analysis of the selected DEPs between the control groups and diclofenac‐treated groups. D) Expression changes of selected DEPs were further validated using parallel reaction monitoring, which showed consistent trends with the SWATH quantitative results. E) Structural model of PBP2a protein complexed with diclofenac compound. In the close‐up view, the hydrogen bonds formed between the compound (magenta) and the protein are depicted as dashed black lines, and the residues involved in the hydrogen bond formation include Thr165 and Glu239. F) Schematic diagram of the possible mechanism employed by diclofenac to resensitize MRSA to *β*‐lactams. Diclofenac reduced the expression of *β*‐lactamase as well as peptidoglycan synthesis and PBP2a activity.

To further validate the antibacterial mechanism of diclofenac, SWATH‐MS and parallel reaction monitoring (PRM) were performed to investigate proteomic alterations in MRSA. SWATH‐MS is a recently developed, quantitative proteomics‐based MS method that combines data independent acquisition (DIA) and targeted data analysis.^[^ ^30^ ^]^ A total of 425 differentially expressed proteins (DEPs) were identified between the diclofenac treatment group and control group, among which 214 were downregulated and 211 were upregulated (**Figure** **6**A). The mass spectrometry proteomics data have been deposited to the ProteomeXchange Consortium with the dataset identifier PXD023055. Next, the functions of these DEPs were further examined by KEGG pathway enrichment analysis (Figure 6B). The results show that diclofenac significantly affected pathways associated with *β*‐lactam resistance, peptidoglycan biosynthesis, and energy metabolism, which is consistent with the RNA‐seq and qRT‐PCR results. Then, the alterations of several DEPs were visualized using a heat map (Figure 6C). After diclofenac treatment, DEPs involved in *β*‐lactam resistance, such as mecR and blaZ, were significantly downregulated. Next, 40 proteins were selected as target proteins for PRM analysis to validate the authenticity and accuracy of the SWATH quantitative results. We found that the expression levels of all target proteins determined by PRM showed consistent trends with the SWATH quantitative results (Figure 6D). The results based on the quantitative methods such as RNA‐seq, qRT‐PCR, SWATH‐MS, and PRM have good consistency, which strongly indicated the reliability of diclofenac to resensitize MRSA to *β*‐lactams. Next, molecular docking was performed to explore whether diclofenac could directly interact with PBP2a to inhibit its activity. Computer‐simulated docking, using a grid box surrounding the entire crystal structure of PBP2, predicted that diclofenac could fit into pockets near Glu239 and Thr165 (Figure 6E), which was found to be important for PBP2a enzyme activity.^[^ ^31^ ^]^ Taken together, the possible mechanism by which diclofenac may resensitize MRSA to *β*‐lactams is presented in Figure 6F.

### 2.7 Diclofenac at its MIC Demonstrates Acceptable Safety in Mammalian Cells

The viability of the cultured cells was evaluated using the CCK‐8 assay and LIVE‐DEAD staining. The CCK‐8 assay demonstrated a dose‐dependent toxic effect with increasing concentrations of diclofenac on cells (Figure S2A in the Supporting Information). As we can see, cell viability was retained above 80% at all concentrations below 125 µg mL^−1^. However, significant toxicity was observed when cells treated with diclofenac at concentrations above 250 µg mL^−1^. Moreover, most cells in the treated groups exhibited similar viability levels as the control groups (Figure S2B in the Supporting Information). This low cytotoxicity of diclofenac at MIC value agrees with the result from the CCK‐8 cytotoxicity test. Besides, compared with the untreated group, cells did not show significant morphological changes characteristic of cell death, including cell shrinkage and few cellular extensions (Figure S2C in the Supporting Information).

### 2.8 Diclofenac Combined with Oxacillin Ameliorates Murine Skin and Soft Tissue Infections

**Figure 7:**
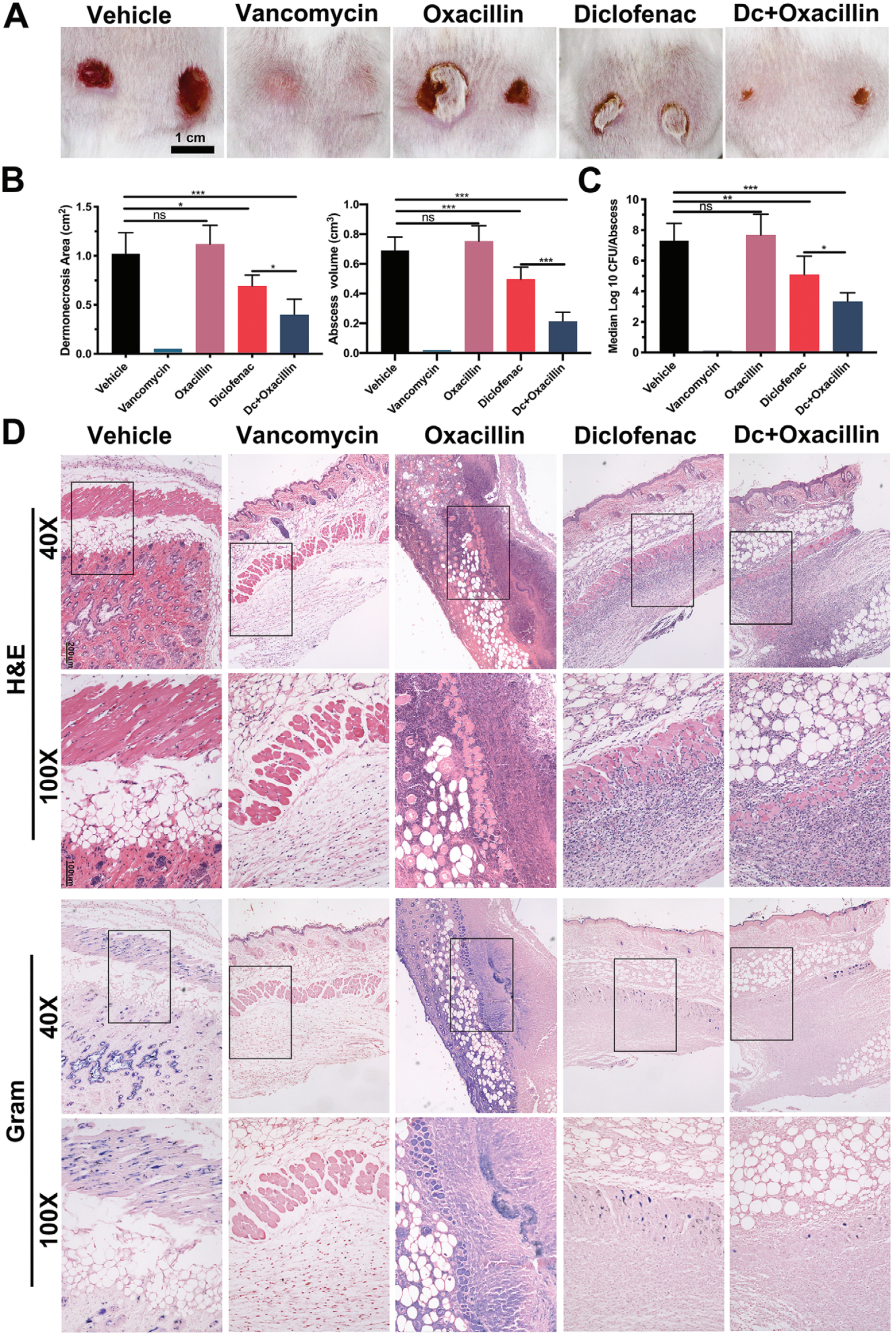
Murine skin and soft tissue infections treated with diclofenac (80 mg kg^−1^, s.c.), oxacillin (200 mg kg^−1^, i.p.), or diclofenac (80 mg kg^−1^, s.c.) combined with oxacillin (200 mg kg^−1^, i.p.). A) Restriction of the abscess area on day 7 in the infection model is shown. Each image shows dermonecrotic lesions from a representative mouse for each group. B) Dermonecrosis areas and abscess volume were measured at selected time points on day 7 in the infection model. Data are expressed as the mean ± SD; *n* = 10; *p*‐values are calculated using one‐way ANOVA with Dunnett correction, *ns*, *not significant*; * *p* < 0.05; *** *p* < 0.001. C) Distribution of CFU density in the abscesses was determined by quantitative culturing from day 7 of the infection. Data are expressed as the means ± SD; *n* = 10; *p*‐values are calculated using one‐way ANOVA with Dunnett correction, *ns*, *not significant*; * *p* < 0.05; ** *p* < 0.01. *** *p* < 0.001. D) Abscess histopathology on day 7 in the infection model. Gram staining as well as hematoxylin and eosin (H&E) staining revealed the location of abscesses and bacteria. In gram‐stained tissues, MRSA cells are dyed violet.

Diclofenac efficacy was analyzed by multiple parameters, including dermonecrotic area, abscess volume, CFU density in situ, and histopathology. As shown in **Figure** **7**A,B, combination of low dose diclofenac and oxacillin significantly restricted the dermonecrosis area and abscess volume compared with that in the vehicle group after 7 days post‐infection with MRSA ATCC 43300 strain. In addition, a single dose of diclofenac resulted in a smaller skin lesion area compared to that in the vehicle group, whereas oxacillin‐treated mice exhibited morphology similar to that in the vehicle group. Furthermore, the number of CFUs within the abscesses was significantly reduced in mice treated with diclofenac compared with that in the vehicle groups (Figure 7C). Lastly, hematoxylin and eosin (H&E) and Gram straining revealed multiple sites of MRSA abscess formation in the vehicle group (Figure 7D). However, mice treated with low dose diclofenac and oxacillin had substantially fewer MRSA micro abscesses and minimal detectable invasion.

### 2.9 Diclofenac Combined with Oxacillin Ameliorates Murine Implant Infections

**Figure 8:**
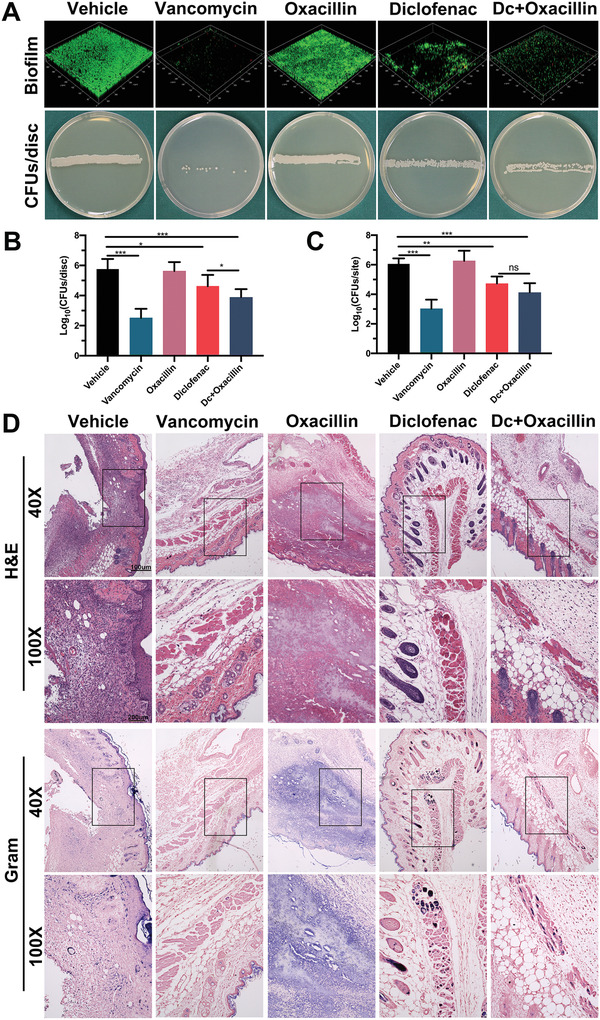
Murine implant infections treated with diclofenac (80 mg kg^−1^, s.c.), oxacillin (200 mg kg^−1^, i.p.), or diclofenac (80 mg kg^−1^, s.c.) combined with oxacillin (200 mg kg^−1^, i.p.). A) Confocal laser scanning microscopy and spread plate method used to observe biofilm formation on the surface of Ti6Al4V disks after 7 days of infection. B) Distribution of CFU density per disk was determined by quantitative culturing from day 7 of the infection. Data are expressed as the means ± SD; *n* = 10; *p*‐values are calculated using one‐way ANOVA with Dunnett correction, * *p* < 0.05; *** *p* < 0.001. C) Distribution of CFU density per site was determined by quantitative culturing from day 7 of the infection. Data are expressed as the means ± SD; *n* = 10; *p*‐values are calculated using one‐way ANOVA with Dunnett correction, *ns*, *not significant*; ** *p* < 0.01, *** *p* < 0.001. D) Histopathological evaluation on day 7 in the infection model. The location of abscesses and bacteria were examined by Gram and hematoxylin and eosin (H&E) staining. In gram‐stained tissues, MRSA cells are dyed violet.

The biofilm‐associated infections were examined by CLSM, spread plate methods, and histopathology. As shown in **Figure** **8**A,B, diclofenac alone or in combination with oxacillin could significantly reduce biofilm formation on Ti6Al4V disks 7 days post‐infection with the MRSA ATCC 43300 strain. Furthermore, the combination of diclofenac and oxacillin exhibited superior antibacterial effects against biofilm‐associated infections. Additionally, the number of CFUs per site was significantly reduced in mice treated with diclofenac compared with that in the oxacillin groups (Figure 8C). Lastly, H&E and Gram straining indicated that mice treated with diclofenac alone, or in combination with oxacillin, had fewer MRSA micro abscesses than mice in the vehicle group (Figure 8D). Taken together, the combination of diclofenac and oxacillin not only reduced the required dose of a single drug but also enhanced the antibacterial effect of *β*‐lactams in the treatment of implant infections.

## 3 Discussion

Approximately one fifth of all annual deaths occur as a result of infectious diseases.^[^ ^13^ ^]^ Moreover, the prevalence of drug‐resistant bacteria and the shortage of available antibiotics have complicated anti‐infective therapy.^[^ ^32^ ^]^ Therefore, drug repurposing screens and synergistic drug combinations have become an effective substitute for the treatment of infectious diseases.^[^ ^33^ ^]^ Compared with administration of single antibiotics, that of synergistic combinations of two known compounds have several advantages. For example, they can reduce the concentrations required for each individual drug, thereby reducing the risk of drug toxicity and medical expenses. Besides, lower concentrations of antibiotics are less likely to induce drug resistance mutations in sensitive bacteria. In this study, we demonstrated that diclofenac, a widely used NSAID, could resensitize MRSA to *β*‐lactams both in vitro and in vivo. In addition, even after continuous exposure to high concentrations of diclofenac, MRSA strains did not develop resistance. Moreover, RNA‐seq and SWATH‐MS results indicated that diclofenac effectively reduced the expression of genes and proteins associated with *β*‐lactam resistance, peptidoglycan biosynthesis, biofilm formation, and bacterial virulence. As a result, the antibacterial efficacy of oxacillin was significantly improved, which might greatly reduce the current dependence on glycopeptide antibiotics such as vancomycin and teicoplanin.

Biofilm formation on the surfaces of implants is another significant challenge associated with treating device‐related infections. Once a biofilm forms on the surface of indwelling devices, it eventually leads to chronic infection.^[^ ^8^, ^34^ ^]^ Even if antibiotics are used at concentrations 100 to 1000 times higher than the normal dose, it is difficult to completely eradicate the biofilm.^[^ ^35^ ^]^ Here, we demonstrated that, the combination of low‐dose diclofenac and oxacillin could effectively inhibit the adherence of bacteria to the surface of titanium disks both in vitro and in vivo. Therefore, this synergistic effect might greatly reduce the probability of implant infections caused by MRSA. RNA‐seq and SWATH‐MS results further indicated that diclofenac not only inhibited the expression of surface proteins associated with biofilm formation but also reduced the production of virulence proteins such as sarR. Therefore, the combination of low dose diclofenac with *β*‐lactams might represent a promising regime for the prevention of biofilm‐associated infections during the perioperative period.

**Figure 9:**
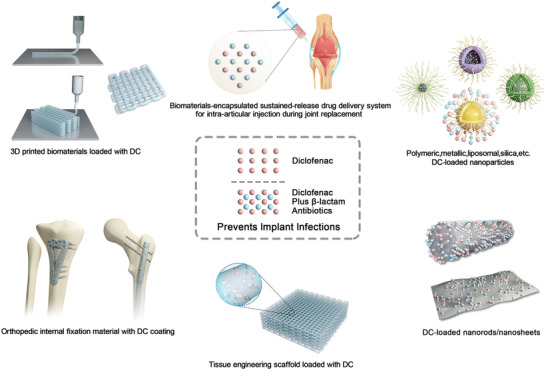
Schematic representation of the potential applications of diclofenac‐loaded biomaterials in the treatment of implant infection caused by MRSA.

In the last few decades, commercial launching of new antibacterial agents has decreased due to difficulties associated with drug discovery and development.^[^ ^9b,c^ ^]^ Therefore, drug repurposing and combination screens may provide alternative approaches at a fraction of the time and cost required for traditional methods of drug development.^[^ ^12^ ^]^ Moreover, clinical trials will require less time to complete as the corresponding pharmacological and safety profiles have already been established for each of the individual drugs. To date, many FDA approved drugs, such as statin and metformin, have been found to have additional antibacterial properties to treat severe infections.^[^ ^14^, ^36^ ^]^ Diclofenac, one of the most common anti‐inflammatory and analgesic drugs, is often used to relieve various kinds of pain, such as postoperative pain. According to research, periarticular injection of large doses of local anesthetics after total hip/knee arthroplasties provides better pain relief and more rapid rehabilitation.^[^ ^23b^ ^]^ Moreover, steroids, NSAIDs, and morphine are often administered in combination with local anesthetics.^[^ ^23^, ^37^ ^]^ Therefore, the addition of diclofenac to the intra‐articular cocktail analgesic injection will not only provide effective pain relief but may also prevent biofilm formation during the early stages of implant infection (**Figure** **9**). Furthermore, the synergistic combinations of diclofenac and *β*‐lactams can increase therapeutic efficacy against MRSA, which could further reduce the need for glycopeptide antibiotics. Therefore, to improve the antibacterial efficiency of diclofenac while simultaneously reducing its side effects, the diclofenac and *β*‐lactam mixture could be loaded on different kinds of biomaterials, such as nanoparticles, nanosheets, and others. (Figure 9).

Although many experiments have been performed to demonstrate the ability of diclofenac to resensitize MRSA to *β*‐lactams, certain gaps in knowledge remain that require further investigation. First, it is important to ascertain the potential adverse side effects associated with consuming oral diclofenac for an extended period of time.^[^ ^38^ ^]^ Hence, it is also necessary to develop a suitable drug delivery system to improve the antibacterial effects while reducing its side effects. Second, investigation into the efficacy of combined therapy comprising diclofenac and other types of antibiotics, such as aminoglycosides, is required, which will be a focus of our future studies.

## 4 Conclusion

In conclusion, diclofenac, one of the most commonly used NSAIDs, was found to effectively inhibit the growth of drug‐resistant staphylococci both in vitro and in vivo. Moreover, high dose diclofenac treatment does not cause drug resistance mutations after continuous passage. Moreover, diclofenac can reduce the expression of genes and proteins associated with *β*‐lactam resistance, peptidoglycan biosynthesis, and biofilm formation, which may help circumvent the development of drug resistance and improve the antibacterial efficacy of conventional antibiotics. Importantly, low dose diclofenac combined with oxacillin, also significantly alleviates MRSA infection in vivo. Hence, this combinatorial therapy may be a promising regime for the prevention of perioperative infections.

## 5 Experimental Section

##### Bacterial Strains and Growth Conditions

MSSA strain ATCC 25923, MRSA strain ATCC 43300, and MRSE strain ATCC 35984 were purchased from the American Type Culture Collection. Bacteria were grown in trypticase soy broth (TSB; BD Biosciences, Franklin Lakes, NJ, USA) overnight at 37 °C with shaking, diluted 1:10 000, and subsequently allowed to reach log phase growth.

##### Antimicrobial Agents and Chemicals

Vancomycin and oxacillin were purchased from Sigma‐Aldrich (St Louis, MO, USA). Diclofenac and daptomycin were purchased from Dalian Meilun Biotechnology Co., Ltd (Dalian, China). All drugs were made up to10 mg mL^−1^ stocks in DMSO or ddH2O. For assays with daptomycin, the medium or buffer was supplemented with 50 µg mL^−1^ CaCl_2_.

##### Minimal Inhibitory Concentration (MIC) Assay

The MICs of the compounds used in this study were determined by the standard microdilution method according to the procedures outlined by the Clinical and Laboratory Standards Institute.^[^ ^39^ ^]^ Briefly, bacteria were grown to log phase and resuspended to a cell density of approximately 5 × 10^5^ CFU mL^−1^. Next, a two‐fold serial dilution of the compound ranging from 500 µg mL^−1^ to 0.98 µg mL^−1^ was prepared in a final volume of 100 µL. All plates were covered and incubated at 37 °C for 24 h. The MIC was taken as the lowest concentration of the agent that completely inhibited visible bacterial growth. All MIC determinations were carried out in duplicate in three independent experiments.

##### Time Kill Assay

The kill kinetics of diclofenac against MSSA ATCC 25923, MRSA ATCC 43300, and MRSE ATCC 35984 was tested as previously described.^[^ ^40^ ^]^ Briefly, 50 µL of diclofenac was serially diluted two‐fold across a 96‐well assay block. Next, 50 µL of bacterial inoculum (5 × 10^5^ CFU mL^−1^) was added and incubated at 37 °C. At specific time points, serial dilutions of the cultures were plated and incubated overnight at 37 °C. The number of viable CFU mL^−1^ remaining in the original culture was calculated by bacterial colony counting.

##### Resistance Induction Assay

Antibiotic‐resistant mutants were acquired by serial passaging over a 20‐day period as described previously.^[^ ^41^ ^]^ Briefly, 100 µL of MRSA ATCC 43300 culture (10^5^ CFU mL^−1^) was added to a 96‐well plate containing an extended concentration gradient of diclofenac, daptomycin or vancomycin. After incubating at 37 °C for 24 h, the OD 600 nm was measured with a spectrophotometer (BioTek, VT, USA). Bacterial growth was defined as OD 600 nm ≥ 0.1. For the following day's MIC plate, 10 µL of the sub‐lethal MIC MRSA cultures were diluted in 10 mL of TSB and incubated overnight at 37 °C. The remainder of the culture was stored in 20% glycerol at ‐80 °C.

##### RNA Isolation and Quantitative RT‐PCR (qRT‐PCR)

An RNeasy mini kit (Qiagen, Frankfurt, German) was used to extract total RNA from MRSA ATCC 43300, according to the manufacturer's instructions. A total of 1 µg of purified RNA was used for cDNA synthesis using the RT Master Kit (Takara, Shiga, Japan). Primers (Sangon Biotech, Shanghai, China) used for the qRT‐PCR reaction are shown in Table S1 in the Supporting Information. A bulk PCR reaction mixture was prepared using the TB Green Premix Ex Taq™ Kit (Takara, Shiga, Japan), and the thermal cycling parameters were as follows: initial denaturation at 95 °C for 30 s; followed by 40 cycles at 95 °C for 5 s, 60 °C for 30 s, and 72 °C for 45 s. Quantitative RT‐PCR was performed in triplicate and repeated in at least five separate experiments using ABI Prism 7500 (Applied Biosystems, Norwalk, CT, USA). Data were analyzed using the comparative CT method, with 16s rRNA expression serving as a normalizer. The results are presented as fold‐changes relative to the control, which was set to a value of 1.

##### RNA Sequencing

Gene expression analysis of the MRSA ATCC 43300 strain was analyzed after treatment with diclofenac (62.5 µg mL^−1^) or 0.1% DMSO. Samples were collected after 8 h of treatment, and three independently prepared RNA samples from each condition were used for RNA Sequencing. Illumina sequencing was performed by Shanghai Majorbio Bio‐pharm Technology Co., Ltd. (Shanghai, China) using the Illumina TruSeq RNA sample prep kit and HiSeq 4000 SBS kit (Illumina, Inc.). After sequencing, the data were analyzed using edgeR software, and statistical significance was defined at *p* < 0.05. Only genes that were significantly differentially regulated (*p* < 0.05) by at least two‐fold compared to the control were considered.

##### Antibiotic Synergy Test

The antimicrobial activity of combined low dose diclofenac and oxacillin was determined through a checkerboard assay as described previously.^[^ ^42^ ^]^ Briefly, two‐fold serial dilutions of diclofenac were combined with two‐fold serial dilutions of oxacillin, creating a 7 × 7 matrix in a 96‐well microtiter plate. After incubating at 37 °C for 18 h, the optical density of each well was determined at 600 nm with a spectrophotometer (BioTek, VT, USA). The FICI of the two compounds (A and B) was calculated as follows: FICI = (MIC of compound A in combination/MIC of compound A alone) + (MIC of compound B in combination/MIC of compound B alone). The interaction between the two compounds was defined as follows: 1) FICI ≤ 0.5 indicates synergy; 2) 0.5 < FICI ≤ 4 indicates “no interaction;” 3) FICI > 4 indicates antagonism.^[^ ^43^ ^]^

##### Transmission Electron Microscopy

Preparation and examination of diclofenac‐treated cells by TEM was performed as described previously.^[^ ^44^ ^]^ Briefly, bacterial cultures were exposed to diclofenac (125 µg mL^−1^), a combination of low dose diclofenac (31.25 µg mL^−1^) and oxacillin (250 µg mL^−1^), or 0.1% DMSO (control) for 8 h at 37 °C. After centrifugation (5 000 rpm; 5 min), the pellets were resuspended in 1 mL of 2.5% glutaraldehyde (Solarbio, Beijing, China). Fixed cells were washed three times with 0.1 M sodium cacodylate buffer and post‐fixed with 1% osmium tetroxide. The samples were stained with 2% uranyl acetate and infiltrated with Epon resin (ProSciTech, Townsville, Australia). Micrographs of the cells were examined by a JEM 1011 TEM (JEOL, Tokyo, Japan).

##### Membrane Permeability Assay

The integrity of the cell membrane was evaluated as described previously.^[^ ^45^ ^]^ Briefly, black 384‐well polystyrene plates (Corning, CLS3573, NY, USA) were filled with 25 µL of PBS per well containing the indicated concentration of compounds. Log phase bacteria were washed three times with PBS and adjusted to 1×10^8^ CFU mL^−1^ with PBS. Then, PI was added to 10 mL of diluted bacterial suspension to a final concentration of 5 µg mL^−1^ and incubated at 37 °C for 30 min in the dark. A total 25 µL volume of the bacteria/PI mixture was added to each well of 384‐well plates containing diclofenac. Lastly, fluorescence was measured using a spectrophotometer (Synergy H1, BioTek), with excitation and emission wavelengths of 535 and 620 nm, respectively. Data were corrected by subtraction of fluorescence signal from untreated cells in the presence of PI. All experiments were conducted in triplicate.

##### Membrane Depolarization Assay

Bacterial cytoplasmic membrane depolarization was detected using the fluorescent dye 3,3‐dipro‐ pylthiacarbocyanine diSC3(5) (Sigma‐Aldrich, Australia) as previously described.^[^ ^40^ ^]^ Briefly, log phase cells were pelleted, washed, and adjusted to an OD600 of 0.2 in assay buffer (10 × 10^−3^ m HEPES, 50 µg mL^−1^ CaCl_2_, and 5 × 10^−3^ m glucose). Next, diSC3(5) was added to a final concentration of 1.5 µM and incubated in the dark to enable dye uptake and a stable reduction in fluorescence. After 30 min, cells were diluted in assay buffer, and 100 µL of bacterial suspension was added to a 96‐well black‐walled plate. Before adding test agents, background data were collected using a spectrophotometer (excitation/emission = 612/665 nm) to ensure fluorescence quenching. Each sample was tested in triplicate, and independent assays were performed three times, showing similar results.

##### Cellular ATP Level Assay

The assay was performed using a BacTiter‐Glo microbial cell viability assay kit (Promega, Madison, WI, USA) as described previously.^[^ ^46^ ^]^ Briefly, log‐phased cells were diluted to 10^6^ CFU mL^−1^ and subsequently incubated with diclofenac or oxacillin. TSB broth without added compounds served as the positive control. After 24 h incubation at 37 °C, bacteria medium was transferred into a black, 384‐well microplate (Corning, CLS3573, NY, USA), and each test sample was mixed with an equal volume of BacTiter‐Glo reagent, which measures the cellular ATP level. The mixed samples were then incubated for 5 min at room temperature, and their luminescence intensities were determined by a Synergy H1 hybrid reader (BioTek, USA) at an integration time of 1 s per well. Relative luminescence units (RLU) values were subtracted from the background control of medium with bacteria.

##### Ethidium Bromide Efflux Inhibition Assay

The efflux pump activity was examined by monitoring the fluorescence of ethidium bromide as previously described.^[^ ^47^ ^]^ Briefly, log‐phased bacterial suspension was pelleted, washed, and resuspended in phosphate buffer saline to an OD600 of 0.2. Next, a sub‐inhibitory concentration of ethidium bromide, the substrate of the efflux pump, was added to a final concentration of 2 µg mL^−1^. The cultures were then incubated for 30 min at 37 °C in the presence of 1/2 MIC diclofenac (62.5 µg mL^−1^) or positive control reserpine (20 µg mL^−1^). Subsequently, 100 µL of the suspensions were inoculated into a black walled 96‐well plate, and the fluorescence was monitored for 30 min with excitation and emission wavelengths of 530 and 600 nm, respectively, using a spectrophotometer.

##### Confocal Laser Scanning Microscopy

Cells exposed to diclofenac (125 µg mL^−1^), a combination of low dose diclofenac (31.25 µg mL^−1^) and oxacillin (250 µg mL^−1^), or 0.1% DMSO were imaged by CLSM. Briefly, bacterial cultures were inoculated in sterile 24‐well plates containing tested compounds. Sterile Ti6Al4V disks were then immersed into the medium as a substratum for biofilm growth and incubated at 37 °C for 24 h. Planktonic cells were removed by rinsing three times with PBS, and bacterial viability was determined using a bacterial viability kit (LIVE/DEAD BacLight, MA, USA). After incubating for 15 min in the dark, the stained disks were observed using a CLSM microscope (Leica TCS SP2; Heidelberg, Germany).

##### Scanning Electron Microscopy

For SEM, the samples were processed as described previously.^[^ ^48^ ^]^ Briefly, exponential‐phase bacterial cultures were grown in 24‐well plates in the presence of sterile Ti6Al4V disks. Then, diclofenac (125 µg mL^−1^), a combination of low dose diclofenac (31.25 µg mL^−1^) and oxacillin (250 µg mL^−1^), or 0.1% DMSO was added to the TSB medium and incubated overnight at 37 °C for 24 h. Next, Ti6Al4V disks were removed and gently washed three times with PBS. The biofilms were fixed with 2.5% glutaraldehyde (Solarbio, Beijing, China) for 24 h at 4 °C. Subsequently, the biofilms were dehydrated for 10 min in an ethanol series (30%, 40%, 50%, 60%, 70%, 80%, and 90%) and for 20 min in absolute alcohol. The samples were observed with a scanning electron microscope (Hitachi S‐4800, Japan).

##### Biofilm Inhibition Assay

The inhibitory effect of diclofenac on biofilm formation was examined using a previously described protocol with several modifications.^[^ ^49^ ^]^ Specifically, 12‐mm diameter Ti6Al4V disks were placed at the bottom of each well of a 24‐well plate (Corning, NY, USA). Next, 2 mL of bacterial culture was added to each well containing the test compound. After incubation at 37 °C for 24 h, planktonic bacteria were removed, and biofilms were stained with 1% crystal violet (Solarbio, Beijing, China) for 15 min. The Ti6Al4V disks were then washed three times with distilled water, and bound dye was extracted from the stained cells using 96% ethanol. Biofilm formation was then quantified by measuring the absorbance of the solution at 595 nm with a microtiter plate reader.

##### SWATH Data‐Independent Acquisition Mass Spectrometry

Quantitative proteomic analyses were performed on proteome extracts of the different MRSA ATCC 43300 samples. Bacteria from three biological replicates were harvested by centrifugation (10 000 × *g*, 10 min, 4 °C) when the OD600 reached 0.6. Next, an Easy‐nLC 1200 system coupled to a Q Exactive HF mass spectrometer (Thermo Fisher Scientific, Bremen, Germany) was employed. Mass spectrometry experiments were conducted according to a label‐free strategy in SWATH DIA mode. For MS1, 3e6 ions were accumulated in the Orbitrap cell over a maximum injection time of 100 ms and scanned at a resolution of 120 000 full width at half maximum from 350 to 1650 *m*/*z*. SWATH spectra were identified by comparison to a reference spectral library obtained with traditional data‐dependent acquisition experiments. Proteins with a fold change equal 1.2 and a *p* value < 0.05 were considered as DEPs. The DEP functions were analyzed based on functional information provided by the Uniprot database (http://www.uniprot.org/).

##### Parallel Reaction Monitoring Assay

Protein extraction and tryptic digestion were conducted as previously described for the SWATH.^[^ ^50^ ^]^ Forty target proteins for PRM validation were analyzed on an Easy‐nLC 1200 system coupled to a Q Exactive HF mass spectrometer (Thermo Fisher Scientific, Bremen, Germany). A scheduled PRM method consisting of full MS1 scan and targeted MS2 scans was developed. The full MS1 scan was collected from 350 to 1650 *m*/*z* at a resolution of 120 000 (AGC target: 3e6, maximum injection time: 100 ms). Targeted MS2 scans were collected from 200 to 2000 *m*/*z* at a resolution of 30 000 (AGC target: 1e5, maximum injection time: 80 ms). Precursors were isolated within a 1.2 *m*/*z* window and fragmented with a normalized collision energy of 27. The peptides monitored for each protein were imported to the Skyline software, and the peptides for protein quantification were selected according to the ion signals in spectra library. Data processing was performed in Skyline, and the quantification results were manually inspected for each peptide of the targeted proteins.

##### Molecular Docking

The crystal structure of *Staphylococcus aureus* penicillin‐binding protein 2a (PBP2a, PDB ID: 1mws) was prepared using the Protein Preparation Wizard software (Schrödinger, USA), and diclofenac (CAS: 15307‐86‐5) was preprocessed by Ligprep 3.4 software (Schrödinger, USA). The allosteric site formed by residues Arg151, Thr165, Glu239, Ser240, Val256, Pro258, Val277, His293, Met372, and Tyr373 was selected to define and generate the receptor grid. *In silico* docking was subsequently performed using the standard precision scoring function of Glide 5.5 software (Schrödinger, USA). The best generated models with docking score −3.96 kcal mol^−1^ for PBP2a were chosen for interaction analysis. All figures for the structure models were generated using UCSF Chimera software.

##### Cytotoxicity Assay

Rat bone marrow mesenchymal stem cells (RBMSC) were identified and cultivated as described previously.^[^ ^51^ ^]^ Compound cytotoxicity was evaluated against MC3T3‐E1 (ATCC CRL‐2594), RBMSC, and human keratinocytes (HaCaT) using both the Cell Counting Kit‐8 (Dojindo Molecular Technologies, Kumamoto, Japan) and the Viability/Cytotoxicity Kit for mammalian cells (Invitrogen, CA, USA). Briefly, 100 µL of RBMSC, MC3T3‐E1, and HaCaT cells were seeded at 1.0 × 10^4^ per well in a 96‐well plate and incubated for 24 h at 37 °C, 5% CO_2_ to allow them to attach to the plates. Next, a two‐fold serial dilution of the compounds to be tested, ranging from 500 µg mL^−1^ to 1.95 µg mL^−1^ was added to the cells and incubated for 24 h. Subsequently, 10 µL of the CCK‐8 reagent was added to each well and incubated for an additional 2 h at 37 °C and 5% CO_2_, and the absorbance of the solution was read at 450 nm using a spectrophotometer (BioTek, VT, USA). In parallel, cell viability was also evaluated with a viability/cytotoxicity kit (Invitrogen, CA, USA) according to the manufacturer's recommendations. Briefly, cells were treated with diclofenac (125 µg mL^−1^) for 24 h, washed twice with PBS buffer, and treated with the viability/cytotoxicity reagent for 45 min at 37 °C. Labeled cells were analyzed by fluorescence microscopy (Nikon, Japan). Viable (live) cells were stained with calcein‐AM (green), whereas dead cells were stained with ethidium homodimer‐1 (EthD‐1; red).

##### Cell Morphology Assessment

The morphology of MC3T3‐E1, RBMSC, and HaCaT cells was evaluated with actin staining with FITC‐labeled phalloidin (Solarbio, Beijing, China). Briefly, after a 24‐h treatment with diclofenac, cells were fixed for 15 min with 4% paraformaldehyde and permeabilized for 5 min in 1% Triton X‐100. Cells were then washed three times with PBS buffer and incubated with a 1:500 dilution of the FITC‐labeled phalloidin for 1 h in the dark. Nuclear DNA was counterstained with DAPI, and stained cells were visualized using a confocal laser scanning microscope (Leica TCS SP2; Heidelberg, Germany).

##### Murine Skin and Soft Tissue Infection Model

All in vivo animal experimental procedures were approved and conducted in accordance with the guidelines of the Animal Ethics Committee of Renji Hospital, China. We followed a previously described protocol to mimic skin and soft tissue infections with some modifications.^[^ ^52^ ^]^ Briefly, eight‐week‐old female CD1 ICR outbred mice (25–30 g) were obtained from Shanghai JieSiJie Laboratory Animal Co., Ltd and randomly divided into five groups: 1) vehicle, 2) vancomycin, 3) oxacillin, 4) diclofenac, and 5) diclofenac plus oxacillin. To induce dermonecrotic infection, exponential‐phase MRSA ATCC 43300 suspension was mixed with an equal volume of autoclaved Cytodex 3 microcarrier beads (Sigma, St. Louis, MO) in PBS buffer. Microbeads were used to ensure localized and uniform lesions in dermonecrotic infections. Each mouse was anesthetized with ketamine and xylazine, and 0.2 mL of the suspension was injected subcutaneously into each shaved flank (1 × 10^6^ CFU per abscess). After 2 h of infection, a single dose of vancomycin (80 mg kg^−1^, i.p.), oxacillin (200 mg kg^−1^, i.p.), diclofenac (80 mg kg^−1^, s.c.), diclofenac (80 mg kg^−1^, s.c.) + oxacillin (200 mg kg^−1^, i.p.), or saline was administered. Thereafter, the treatment was administered every 24 h until 7 days post‐infection.

##### Abscess Evaluation—Magnitude

After infection, dermonecrosis area and abscess volume were measured in each mouse flank. To quantify dermonecrosis area (cm^2^), lesion site length (*l*) and width (*w*) were measured. Next, abscess volume (cm^3^) was calculated according to the formula for a spherical ellipsoid: [v = (*π*/6) × *l* × *w* ^2^].

##### Abscess Evaluation—CFU Determination

Seven days post‐infection, mice were sacrificed and processed for quantitative culture of abscesses. Each flank was aseptically dissected, and the abscess was removed and prepared for culture. After individually homogenizing and serially diluting the suspensions in sterile PBS, the dilutions were spread onto TSA plates. The plates were incubated at 37 °C for 24 h and the resulting colonies counted as CFU per abscess.

##### Abscess Evaluation—Histological Evaluation

Seven days post‐infection, tissue inflammation and bacterial quantity related to MRSA ATCC 43300 strain invasion were assessed in the skin abscesses. For histological staining, excised skin specimens were fixed in 4% paraformaldehyde and embedded in paraffin. Next, sections of skin tissue were observed under a microscope after H&E, and Gram staining was performed.

##### Murine Implant Infection Model

Implant infection models were established as previously described,^[^ ^53^ ^]^ with several modifications. Briefly, 8‐week‐old specific pathogen‐free (SPF) CD1 ICR female mice were obtained from Shanghai JieSiJie Laboratory Animal Co., Ltd. and randomly divided into five experimental groups: 1) vehicle; 2) vancomycin; 3) oxacillin; 4) diclofenac; 5) diclofenac + oxacillin. Each mouse was anesthetized with ketamine/xylazine; the flanks of mice were shaved, and an incision was made parallel to the spine. Next, sterilized Ti disks were placed into the subcutaneous pocket using aseptic technique and the incision sutured layer by layer. Then, 100 µL of exponential‐phase MRSA ATCC 43300 suspension (10^5^ CFU per site) was injected into the subcutaneous biomaterial bed. After 2 h of infection, a single dose of vancomycin (80 mg kg^−1^), oxacillin (200 mg kg^−1^, i.p.), diclofenac (80 mg kg^−1^, s.c.), diclofenac (80 mg kg^−1^, s.c.) + oxacillin (200 mg kg^−1^, i.p.), or saline was administered. Thereafter, treatment was administered every 24 h until 7 days post‐implantation. To determine the bacterial load and biofilm formation, the Ti disks and soft tissue surrounding the implants were removed from each site and separately immersed in PBS. After individually homogenizing and serially diluting the suspensions, the dilutions were spread onto TSA plates. The plates were incubated at 37 °C for 24 h, and the number of bacteria on the implants or in the tissues were calculated. For histological evaluation, excised skin specimens were fixed in 4% paraformaldehyde and embedded in paraffin. Next, sections of skin tissue were observed microscopically after H&E and Gram staining.

##### Statistical Analyses

The results were analyzed using Prism 7 (GraphPad Software Inc., CA, USA), and the data are presented as the mean ± standard deviation (SD). Statistical significance was determined by unpaired two‐tailed Student's *t* tests where only two groups existed or by one‐way ANOVA with Dunnett's or Tukey's post‐test. Sample size (*n*) and preprocessing normalization of data was given in the corresponding figure legend. Differences between groups were considered significant at p < 0.05. (*p<0.05, **p<0.01, ***p<0.001).

## Conflict of Interest

The authors declare no conflict of interest.

## Supporting information

Supporting Information

## Data Availability

The data that support the findings of this study are available from the corresponding author upon reasonable request.
