# Supplementary material for: Diclofenac Resensitizes Methicillin‐Resistant Staphylococcus aureus to β‐Lactams and Prevents Implant Infections
Source: Adv Sci (Weinh). 2021 May 3;8(13):2100681. doi: 10.1002/advs.202100681 (PMC8261494; doi:10.1002/advs.202100681)
Supplement: Supplementary file 1 — Supporting Information [file ADVS-8-2100681-s001.pdf]

© 2021 Wiley-VCH GmbH

ADVANCED SCIENCE

Open Access

Supporting Information

for *Adv. Sci.,* DOI: 10.1002/advs.202100681

Diclofenac Resensitizes Methicillin-resistant *Staphylococcus aureus* to β-lactams and Prevents Implant Infections

Shutao Zhang, Xinhua Qu, Haozheng Tang, You Wang, Hongtao Yang, Weien Yuan and Bing Yue**

Supporting Information

Diclofenac Resensitizes Methicillin-resistant *Staphylococcus aureus* to β-lactams and Prevents Implant Infections

Shutao Zhang[1#], Xinhua Qu[1#]*, Haozheng Tang[1], You Wang[1], Hongtao Yang[2,3], Weien Yuan[4] and Bing Yue[1]*

Table S1

Primer used in this study

Target gene	direction	Primer sequence (5'to 3')
16s rRNA	Forward	CGCAATGGGCGAAAGC
	Reverse	TACGATCCGAAGACCTTCATCA
murA	Forward	GTGTACGTGCTGAAGGGGAA
	Reverse	GCGGCTGCTGCTCTTAAATC
murC	Forward	TCGGATGACATTTATGCTCAAAA
	Reverse	ACCATCCACATACACATCAAAAGC
femA	Forward	TGCTTTCCGTCATTTTGCCG
	Reverse	ACACCAGCATCTTCAGCATCT
femB	Forward	TCGCATGGTTACGAGCATCA
	Reverse	TCCAGCACGCTCTTCAGTTT
atlE	Forward	GACCCTGCTATTGTCCAACCA
	Reverse	TCGCACGAGTGTCACCATTT
clfA	Forward	GAAAAACACGCAATTCGGAAA
	Reverse	CCAAAACCGATTAACGTACCTACA
fnbP	Forward	CAACAGGTGCTAACCAAGCAA
	Reverse	TGAGACGCTTTATCCTCAGTCG
vraS	Forward	GCGTTCAATGGAAGGCGAAA
	Reverse	TTGCCGCAAAAAGTTGCTGA
vraR	Forward	CGCGCAGAGTTATATGAAATGC

	Reverse	CGCTAATGATTGCGAAGACCT
agrA	Forward	TTGCCCTCGCAACTGATAAT
	Reverse	CAACTGGGTCATGCTTACGA
agrB	Forward	TGACCAGTTTGCCACGTATCT
	Reverse	AGACCTGCATCCCTAATCGT
agrC	Forward	AAATGCGCAAGTTCCGTCAC
	Reverse	GGCCAGGCATGTCATCTTCT
rnaIII	Forward	GGAGTGATTTCAATGGCACAAG
	Reverse	TGTTCACTGTGTCGATAATCCATTT
mecR	Forward	ATGCGAATGGCGAAAAAGCA
	Reverse	TGGACTCCAGTCCTTTTGCAT
mecA	Forward	TGGCAGACAAATTGGGTGGT
	Reverse	TGAAGCAACCATCGTTACGGA
blaZ	Forward	TGCTTTAGTTTTAAGTGCATGT
	Reverse	TCCTTCATTACACTCTTGGC

Table S2

MICs of β-lactams alone or in combination with diclofenac (31.25 μg/ml).

antibiotic	MRSA ATCC 43300	
	Alone MIC (μg/ml)	Combined MIC (μg/ml)
Oxacillin	1000	250
Ampicillin	500	62.5
Penicillin G	500	125
Cefazolin	1000	125
Cefoxitin	500	125

Figure S1

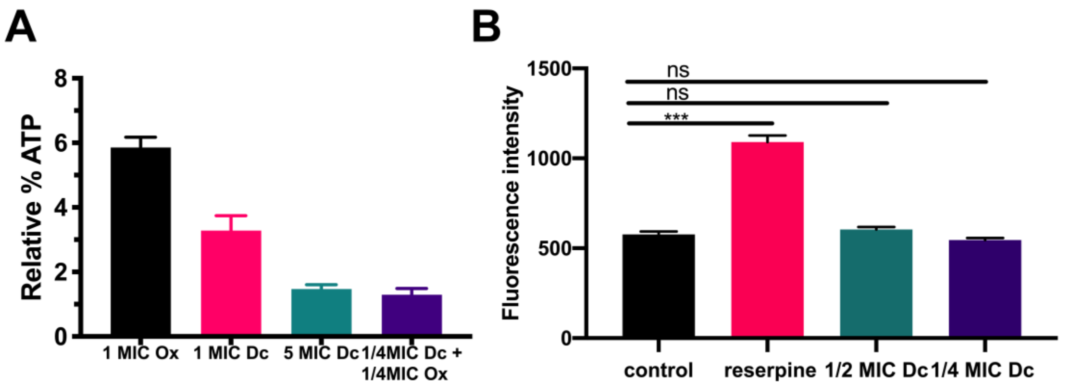

Figure S1. Diclofenac affected the energy metabolism of MRSA. **(A)** Cellular ATP level assay. BacTiter-Glo microbial cell viability assay kit was used to examine the cellular ATP level of MRSA ATCC 43300 after diclofenac or oxacillin treatment. Relative luminescence units (RLU) values were subtracted from the background control of medium with bacteria. **(B)** Ethidium bromide efflux inhibition assay.

Fluorescence-based detection of ethidium bromide efflux by MRSA ATCC 43300 in the presence and absence (control) of diclofenac at 1/2 MIC and 1/4 MIC value. Reserpine was used as a known positive control. This assay was performed in triplicate and results are expressed as the means ± SD. *P*-values are calculated using one-way ANOVA with Dunnett correction, *ns*, *not significant*; ** $p < 0.01$, *** $p < 0.001$.

Figure S2

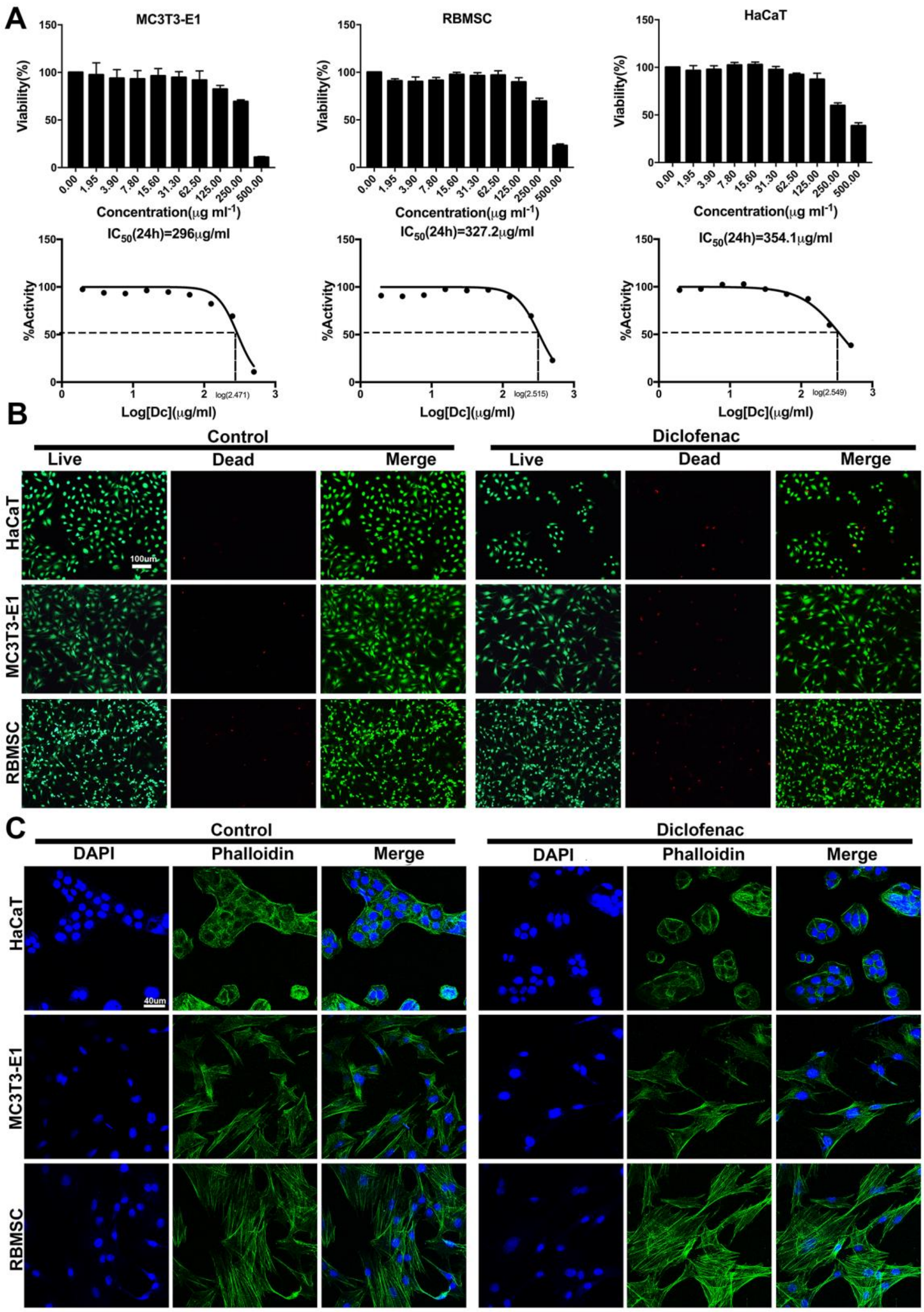

Figure S2. Diclofenac does not affect mammalian cell viability at MIC value. (A) MC3T3-E1 cells, RBMSC, and HaCaT cells were treated with different

concentrations of diclofenac for 24 h, and cell proliferation was tested by CCK-8 assays. The half-maximal inhibitory concentration (IC50) of diclofenac after 24 h was calculated using GraphPad Prism 7.0. (B) Fluorescence microscopy images of MC3T3-E1 cells, RBMSC, and HaCaT cells after 24-h treatment with diclofenac, stained using the Viability/Cytotoxicity Kit for mammalian cells. Living cells are shown stained with calcein-AM (green color) and dead cells are shown stained with ethidium homodimer (EthD-1) (red color). (C) CLSM analysis of the morphology of MC3T3-E1 cells, RBMSC, and HaCaT cells after 24-h treatment with diclofenac. The cells were stained with FITC-labeled phalloidin and DAPI. Experiments were repeated at least three times.
